# Novel Techniques in Antenatal Imaging of Spinal Dysraphisms

**DOI:** 10.1007/s11934-025-01258-4

**Published:** 2025-03-06

**Authors:** Charis Royal, Leon Chertin, Mohammed Alfawzan, Mary Elaine Killian

**Affiliations:** 1 Department of Urology, Division of Pediatric Urology, Le Bonheur Children’s Hospital, 50 N Dunlap Street, Memphis, TN 38103 USA; 2 University of Tennessee Health Sciences Center, 50 N Dunlap Street, Memphis, TN 38103 USA

**Keywords:** Spinal dysraphism, Prenatal ultrasound, Magnetic resonance imaging, Neural tube defects, 3D ultrasound, Spina Bifida

## Abstract

**Purpose of Review:**

This review examines the imaging techniques for diagnosing spinal dysraphisms (SD), focusing on advancements in prenatal detection.

**Recent Findings:**

Prenatal ultrasound (US) is the first-line tool for detecting spinal dysraphisms, including myelomeningocele. While US is effective for early detection, it has limitations in fully characterizing defects, particularly due to factors like fetal positioning. To address these, advanced techniques such as 3D ultrasound and AI-driven algorithms have improved diagnostic accuracy. Magnetic resonance imaging (MRI) remains critical for a comprehensive evaluation, providing detailed visualization of soft tissue anomalies and assessing lesion severity.

**Summary:**

Prenatal ultrasound is essential for initial screening but often complemented by MRI for a thorough diagnosis. Innovations in imaging technologies, including AI and 3D ultrasound, promise to enhance early detection and clinical management of spinal dysraphisms.

## Introduction

Spina bifida and other spinal dysraphisms are significant congenital malformations of the central nervous system, with an estimated global incidence of approximately 1 in 1,000 live births [1]. The prevalence can vary depending on geographic location, with higher rates observed in regions such as North America and Europe, as well as among populations with specific genetic predispositions [1]. Spina bifida, particularly myelomeningocele, is the most common form, and it accounts for approximately 70–90% of all cases of open spinal dysraphism [1, 2]. Other forms, such as tethered cord syndrome or lipomyelomeningocele, are less common but still critical to detect for proper management.

These conditions are most commonly diagnosed during prenatal screening, typically through ultrasound imaging performed during the second trimester [1]. Prenatal ultrasound can identify the presence of neural tube defects, including spina bifida, by visualizing abnormal spinal structures [2, 3]. In some cases, additional imaging techniques, such as magnetic resonance imaging (MRI), are used postnatally to better define the extent of the malformation and to assess associated complications, aiding in surgical planning and prognosis [1].

Spinal dysraphism (SD), which is defined as a wide range of congenital spinal defects that arise from incomplete neural tube closure during embryonic development, exhibits a broad spectrum of severity, ranging from asymptomatic to severely disabling neurological conditions [2]. Spinal dysraphism affects approximately 1 to 3 out of every 1,000 newborns, most commonly in the lumbosacral region [3, 4].

To properly treat these conditions, it is essential to make an accurate diagnosis and determine the severity of the spinal abnormality, however, this is heavily dependent on advanced imaging modalities. Prenatal ultrasound often provides initial detection, while postnatal evaluations frequently utilize magnetic resonance imaging (MRI) and sometimes computed tomography (CT) scans to precisely characterize the type and severity of the spinal defect [2, 5, 6]. These imaging techniques are essential in guiding appropriate management strategies and predicting long-term outcomes for newborns with SD [6, 7].

The purpose of this review is to provide a comprehensive analysis of the diagnostic imaging techniques employed in the detection and assessment of SD.

### Ultrasound

Prenatal ultrasound (US) serves as the primary imaging modality for detecting spinal dysraphisms during pregnancy due to its non-invasive, accessible, and cost-effective nature [2]. As the first-line imaging tool, it is commonly used for the detection of congenital spine malformations, including spina bifida. Ultrasound offers a detailed view of the spinal column and surrounding structures, enabling early detection of major defects such as myelomeningocele and other abnormalities, especially in the second trimester [4]. However, while it is effective for identifying the presence of spinal defects, ultrasound has limitations in fully characterizing the severity of the condition. Factors such as fetal positioning, maternal body habitus, and image quality can obscure the visualization of bone structures and neural components, making it challenging to assess the extent of malformations or associated anomalies [5, 8]. As such, while ultrasound is indispensable for initial screening and early detection, its use is often complemented by advanced imaging techniques like magnetic resonance imaging (MRI) for a more comprehensive assessment of spinal dysraphism, including its location and associated complications [2, 5, 8].

In some instances, clinicians encounter the challenge of detecting spinal structures in ultrasound images of fetuses with spina bifida, where bone tissue may not be clearly visible due to developmental stages. In one study, Cengizler et al. presented a novel method for enhancing the accuracy and speed of prenatal spina bifida diagnosis. This method, which improves fetal spine identification in ultrasound images, shows significant promise for clinical application and holds significant potential for integration into a fully automated computer-aided diagnosis system [9]. The proposed method shows potential for automating spine detection in ultrasound images, which could aid in early diagnosis and treatment planning for spina bifida cases. In a second study by Cengizler et al. expanded on this proposed image processing technique to reduce noise and enhance bone visibility. In this technique, images are resized, converted to grayscale, blurred, and processed into binary masks to isolate bone-like regions [10]. The baseline detection rate for spinal structures is 65%, however, when utilizing the algorithm in this study, the detection rate increased to 90%. Additionally, there was a reduction in false positive readings from 20 to 5% and a reduction in analysis time from 45 min to 15 min.

Barnes et al., assessed the accuracy of prenatal ultrasound in estimating the anatomic level of myelomeningocele (MMC) and its correlation with maternal BMI, involving 57 patients [11]. Results demonstrated moderate to substantial agreement between prenatal ultrasound and postnatal imaging, with 87.7% agreement within two spinal levels, indicating that ultrasound is a reliable diagnostic tool regardless of maternal BMI. These findings support the use of prenatal ultrasound for treatment planning and highlight the importance of accurate imaging for informed family counseling. Additionally, the study suggests that the level of the lesion predicted by ultrasound can significantly influence prognostication and intervention eligibility.

Zhu et al., in their study investigated the effectiveness of cranial ultrasound markers during the first trimester in diagnosing open spina bifida (OSB) [12]. The research focused on specific ultrasound markers: the 3-line view, the brain stem (BS)-to-brain stem-occipital bone (BSOB) distance ratio, the maxillo-occipital (MO) line, and the crash sign. Findings revealed that 87.5% of OSB cases exhibited the 3-line view, while all OSB cases had a BS/BSOB greater than 1 and an abnormal MO line, demonstrating 100% sensitivity for these markers. In contrast, closed spina bifida cases did not present these markers. The study concludes that these ultrasound markers are effective for early detection of OSB, emphasizing the importance of first-trimester scans for timely diagnosis. A spinal ultrasound (US) evaluation during the immediate postnatal period may have limited ability in evaluating filum thickness because of the spinal cord pulsation caused by a crowded subarachnoid space and the cerebrospinal fluid deficiency during this period [12].

A small study suggests a potential link between smaller fetal lateral ventricles on first-trimester ultrasound and open spina bifida, possibly due to reduced ventricular fluid volume; however, these findings require validation in a larger study [13]. Another small study of only five cases suggests a potential correlation between the implementation of a combined cranial and spinal ultrasound approach during the first trimester and increased detection rates for open spina bifida [14].

A study by Cho et al. evaluated the optimal timing for spinal ultrasounds in neonates with sacral dimples, finding that performing the ultrasound within the first month can lead to inaccurate assessments due to spinal pulsation and cerebrospinal fluid deficiencies [15]. Delaying the evaluation until neonates are older than 31 days, with a corrected age over 42.5 weeks and a weight above 4.6 kg, improves diagnostic accuracy and reduces unnecessary costs. Most initially inconclusive cases did not show significant abnormalities upon follow-up, highlighting the importance of careful monitoring. These findings can inform clinical guidelines for managing spinal dysraphism and optimize healthcare resources.

Compared to ultrasound, fetal MRI offers significantly improved accuracy in diagnosing spinal cord malformations, while showing comparable accuracy for purely spinal defects [16]. Conversely, systematic review indicates that current prenatal diagnostic methods, including ultrasound and MRI, exhibit only moderate accuracy (approximately 40–42%) in identifying the precise location of spinal lesions in fetuses with open spina bifida. Further research and development of improved techniques are therefore warranted to enhance the accuracy of prenatal diagnosis [1].

A review of 245 open spina bifida cases suggests that comprehensive initial ultrasound assessment is important for accurate determination of surgical eligibility, as 20% of cases initially considered suitable for fetal surgery proved ineligible upon further review at a specialized center [17].

### Magnetic Resonance and Other Modalities

MRI is recognized as the gold standard for diagnosing spinal dysraphism due to its unparalleled ability to provide detailed images of soft tissues, which are critical in identifying neural tube defects and associated anomalies. Studies highlight the crucial role of MRI in visualizing conditions such as myelomeningocele and Chiari malformations, which often accompany spinal dysraphism [2, 18].

Tawfik et al., investigated the diagnostic accuracy of spinal ultrasonography (USG) compared to magnetic resonance imaging (MRI) in identifying SD. The study involved 45 infants and children, aged from newborn to 12 years, who presented with clinical signs suggestive of SD, such as back swelling, skin dimples, or sacral sinuses [19]. In children aged ≤ 2 years, there was an excellent agreement (κ = 0.96) between USG and MRI findings. In children aged > 2 years, the agreement was fair (κ = 0.58), indicating a decrease in diagnostic concordance as age increased. For patients ≤ 2 years, USG demonstrated high specificity (94.5–100%) and sensitivity (84.3–100%) in detecting SD. The study highlighted that USG is particularly effective in identifying abnormalities in the conus medullaris and filum terminal in younger patients.

Hussein et al., showed similar results in their study of evaluating the effectiveness of USG in diagnosing closed spinal dysraphism (CSD) in infants, and comparing its accuracy with MRI. USG demonstrated high specificity (98.6–100%) and sensitivity (66.6–91.6%) for detecting spinal dysraphism [20]. Another important conclusion from this study that 57.9% of infants with anorectal malformations had associated spinal dysraphism, indicating the importance of screening in this population. USG was particularly effective in cases of complete agenesis of the spine but less so in segmentation anomalies. USG limitations increase after 6 months of age due to ossification of spinal elements.

MRI not only excels in initial diagnoses but also provides essential information for surgical planning. It assists in evaluating the extent of the malformation, helping clinicians make informed decisions about surgical interventions and potential outcomes [16, 21].

### Computed Tomography (CT)

CT scans are often employed as a supplementary imaging tool, particularly when detailed visualization of bony structures is necessary. They can effectively identify osseous anomalies associated with spinal dysraphism, which can inform surgical decisions [22, 23]. However, the use of ionizing radiation is a notable drawback, making CT less desirable for initial evaluations, especially in pediatric populations where minimizing radiation exposure is critical.

### Rapid MRI Techniques

The development of rapid MRI protocols, particularly the T2-weighted Half-Fourier Acquisition Single-shot Turbo spin Echo (T2 HASTE), has significantly improved the ability to perform MRI without sedation in neonates. Khalatbari et al. demonstrated that this rapid imaging technique could be used to screen for spinal cord syrinx in neonates with spinal dysraphism [23]. In this study, out of 26 neonates with myelomeningocele, 5 (19%) were identified with spinal cord syrinx using rapid MRI. This method minimizes the need for anesthesia, reducing associated risks while providing timely diagnostic information. Additionally, this technique greatly reduced the time a neonate would need to be under sedation as well as effectively visualized the spinal anatomy and associated anomalies. These findings are particularly important as they underscore patient safety as well as rapid diagnostics.

### Emerging Imaging Modalities

Recent advancements in imaging techniques continue to emerge such as Magnetic Resonance Spectroscopy (MRS), Three-Dimensional Imaging, and Artificial Intelligence.

#### Magnetic Resonance Spectroscopy (MRS)

MRS can analyze the biochemical composition of spinal tissues, providing insights into metabolic changes related to spinal dysraphism [6]. This could lead to enhanced diagnostic capabilities by revealing underlying pathophysiological processes. Some proponents state that utilizing this biochemical test in concert with rapid imaging techniques could improve diagnosis in neonates with limited complications or risk.

#### Three-Dimensional Imaging

Innovations in 3D imaging techniques allow for comprehensive volumetric assessments, which enhance understanding of spatial relationships within the spinal canal. Such detailed imaging is crucial for accurate evaluations of complex spinal malformations. A study by Huang et al. retrospectively reviewed cases of neural tube defects over an 11-year period and noted that utilization of 3D ultrasound facilitated the detection of additional anomalies not initially seen on 2D ultrasound [6]. These results demonstrate that 3D imaging techniques can improve diagnostic capabilities and aid in in-utero surgical repairs.

#### Artificial Intelligence (AI)

The integration of AI into imaging workflows is an exciting development. Machine learning algorithms can analyze vast amounts of imaging data, helping identify subtle patterns and abnormalities that may be missed by human observers. This could lead to improved diagnostic accuracy and more timely interventions [17]. A study by Drukkar et al. showed that deep-learning based analysis of nearly 500 s-trimester fetal anomaly ultrasound scans demonstrated that sonographer examination sequences are highly variable and driven by the opportunistic use of fetal positioning to maximize visualization, rather than strict adherence to established guidelines. This highlights the potential for the application of artificial intelligence (AI) in optimizing both sonographer training and the overall efficiency of the anomaly scanning process [24]. There is a significant potential for this process to be expanded to MRI sequences and other imaging modalities to increase the ability to accurately diagnose SD.

### Fetal MRI Innovations

Innovations in fetal MRI techniques are evolving to enhance prenatal assessments of spinal dysraphism. These advancements focus on optimizing imaging protocols to ensure safety and accuracy in diagnosing conditions like spina bifida in utero. Enhanced imaging capabilities are crucial for developing effective management strategies, allowing for informed decision-making regarding potential surgical interventions [22, 23].

### Combination Imaging Approaches

Future imaging strategies may increasingly involve combining MRI with other modalities, such as CT or positron emission tomography (PET). This multimodal approach could provide a comprehensive understanding of spinal pathologies, assessing both structural and functional changes, thereby enhancing diagnostic capabilities [22, 23].

### Telemedicine and Remote Imaging Evaluation

The rise of telemedicine has transformed access to specialized imaging interpretations. Remote evaluation of complex imaging studies enables timely consultations, particularly for patients in underserved regions. This integration ensures that patients receive the necessary care without geographical barriers [17].

## Conclusion

The imaging landscape for spinal dysraphism is continually evolving. MRI remains the cornerstone of accurate diagnosis due to its unmatched ability to visualize complex spinal anatomy. Current advancements in MRI technology and novel methodologies enhance diagnostic capabilities, while emerging techniques, including AI and combination imaging, promise to refine and improve patient outcomes. As these innovations develop, they will likely play a pivotal role in the timely and effective management of spinal dysraphism, ultimately improving patient care in both prenatal and postnatal contexts.

## Key References

1. Tawfik NA, Ahmed AT, El-Shafei TE, Habba MR. (2020)– *Diagnostic Value of Spinal Ultrasound Compared to MRI for Diagnosis of Spinal Anomalies in Pediatrics*.

Very Important: This study highlights the high diagnostic accuracy of ultrasound in younger children, showing it as a reliable first-line tool and influencing prenatal and postnatal imaging practices.

2. Cengizler C, Kerem Ün M, Buyukkurt S. (2020)– *A Nature-Inspired Search Space Reduction Technique for Spine Identification on Ultrasound Samples of Spina Bifida Cases*.

Very Important: This paper introduces an innovative, nature-inspired algorithm that significantly improves spine detection accuracy in ultrasound images of spina bifida which could lead to automated solutions for early, accurate diagnosis in prenatal imaging.

3. Zhu X, Zhao S, Yang X, et al. (2021)– *First-Trimester Cranial Ultrasound Markers of Open Spina Bifida*.

Important: This study identifies crucial first-trimester ultrasound markers for open spina bifida, providing high sensitivity for early diagnosis. Jakab A, Varga S, Szabo M, Olasz L, Bartha E. (2021)– *Emerging Magnetic Resonance Imaging Techniques in Open Spina Bifida in Utero*.

Important: This review explores advanced MRI techniques, such as diffusion tensor imaging (DTI) and super-resolution reconstruction, that enhance the evaluation of open spina bifida in utero.

## Data Availability

No datasets were generated or analysed during the current study.
